# Methicillin resistance and virulence genes in invasive and nasal *Staphylococcus epidermidis* isolates from neonates

**DOI:** 10.1186/s12866-017-0930-9

**Published:** 2017-01-13

**Authors:** Vivian Carolina Salgueiro, Natalia Lopes Pontes Iorio, Marcelle Cristina Ferreira, Raiane Cardoso Chamon, Kátia Regina Netto dos Santos

**Affiliations:** 1 Departamento de Microbiologia Médica, Instituto de Microbiologia Paulo de Góes, Universidade Federal do Rio de Janeiro, Av Carlos Chagas Filho, no 373, CCS, Bloco I, Sala 010, Cidade Universitária, Rio de Janeiro, Brazil; 2 Departamento de Ciências Básicas, Universidade Federal Fluminense, R. Dr. Silvio Henrique Braune, no 22, Nova Friburgo, Rio de Janeiro Brazil

**Keywords:** *S. epidermidis*, Neonates, Bloodstream infection, Nasal, SCC*mec*, Virulence

## Abstract

**Background:**

*Staphylococcus epidermidis* is an opportunistic pathogen involved in hospital-acquired infections, particularly in those related to medical devices. This study characterized 50 genetically unrelated *S. epidermidis* isolates from bloodstream infections (BSIs, *n* = 31) and nares (*n* = 19) of neonates in relation to staphylococcal chromosomal cassette *mec* (SCC*mec*) type, biofilm production and associated genes, and the arginine catabolic mobile elements (ACME), in order to detect virulence factors that could discriminate a potential invasiveness isolate or predict an increasing pathogenicity.

**Results:**

Isolates from both groups showed no difference for biofilm production and ACME genes detection. However, BSI isolates harbored more frequently the *sdrF* and *sesI* genes (*p* < 0.05), whereas biofilm producer isolates were associated with presence of the *aap* gene. The *sdrF* gene was also significantly more in the biofilm producer isolates from BSI. The SCC*mec* type IV and the *ccr*2 complex were related to BSI isolates (*p* < 0.05), while 83% of the nasal isolates were non-typeable for the SCC*mec* elements, with the *mec* complex and *ccr* undetectable as the most frequent profile.

**Conclusions:**

Despite the great clonal diversity displayed by *S. epidermidis* isolates from neonates, BSI isolates harbored more frequently the *sdrF* and *sesI* adhesin genes, while nasal isolates were very variable in SCC*mec* composition. These aspects could be advantageous to improve colonization in the host increasing its pathogenicity.

## Background

*Staphylococcus epidermidis,* a common human commensal microorganism that colonizes skin and mucosal surfaces, has become an opportunistic pathogen, due to its ability to colonize invasive medical devices causing bloodstream infections (BSI) [1]. Some of the interventions used to treat neonates, particularly those admitted to neonatal intensive care units (NICUs), including prolonged antibiotic use and invasive procedures that disrupt the skin integrity, may expose neonates to the risk of developing *S. epidermidis* infections [2].

A wide range of surface proteins with adhesive properties improves the ability of *S. epidermidis* to adhere to different surfaces [1]. The Bhp protein (Bap homologue protein) and the autolisin/adhesin AtlE (autolysin of *S. epidermidis*) mediate the initial adhesion through hydrophobic interactions [3]. Almost at the same time, human extracellular matrix components bound and cover the polymeric surface, and a group of microbial proteins called Microbial Surface Components Recognizing Adhesive Matrix Molecules (MSCRAMMs), like SdrF, SdrG (also known as Fbe) and Embp (extracellular matrix-binding protein) can specifically bind to collagen, fibrinogen and fibronectin, respectively [4–6]. Additionally, AtlE and Aae (autolysin/adhesin of *S. epidermidis*) proteins bind nonspecifically to fibrinogen, fibronectin and vitronectin [7, 8]. Other *S. epidermidis* proteins have also been described as putative adhesins, like the GehD lipase that binds to collagen [9] and the *S. epidermidis* surface (Ses) proteins, among them SesI, has gained attention due to their immunogenic properties [10] and its association with invasive isolates [11]. Many *S. epidermidis* isolates carry the *icaADBC* operon that encodes proteins involved in the synthesis of the exopolysaccharide PIA (polysaccharide intercellular adhesin), which connects the bacteria cells in the biofilm [12]. PIA together with Embp, Bhp and Aap (accumulation-associated proteins) are responsible for the intercellular adhesion and accumulation, enabling the biofilm formation [6, 13, 14].

*S. epidermidis* isolates have presented methicillin resistance, which is determined by the acquisition of the *mecA* gene, carried by a genetic mobile element known as staphylococcal chromosomal cassette *mec* (SCC*mec*). The *mecA* gene encodes a modified penicillin-binding protein (PBP2a) that presents low affinity for beta-lactam antibiotics [15]. Eleven types (I to XI) of SCC*mec* have been assigned for *Staphylococcus aureus* based on the classes of the *mec* gene complex and the types of the *ccr* gene complex [16]. In *S. epidermidis* these elements are very diverse and most of the isolates are defined as non-typeable [17–19].

The presence of the arginine catabolic mobile element (ACME) among *S. epidermidis* isolates has been receiving more attention since it may provide advantages in host colonization by staphylococcal cells [20]. This genetic element is composed of two gene clusters, the *arc*-operon, encoding a secondary arginine deiminase system and the *opp3-*operon that encodes a putative oligopeptide permease system [21]. It has been proposed that the *ccr* recombinase of the SCC*mec* element could be responsible by the ACME mobilization, suggesting that the horizontal transfer of these two elements may be linked [20].

Despite the increased number of studies involving the *S. epidermidis* species, there are still few studies that have detected characteristics that could distinguish infection and colonization isolates, especially among isolates from neonates. In this study we evaluated 50 genetically unrelated *S. epidermidis* isolates from bloodstream infections and nasal colonization of neonates in relation to SCC*mec* types, biofilm formation and associated genes, and the presence of ACME in order to detect virulence factors that could distinguish a potential invasiveness isolate or predict an increasing pathogenicity.

## Methods

### Clinical isolates

**Fig. 1:**
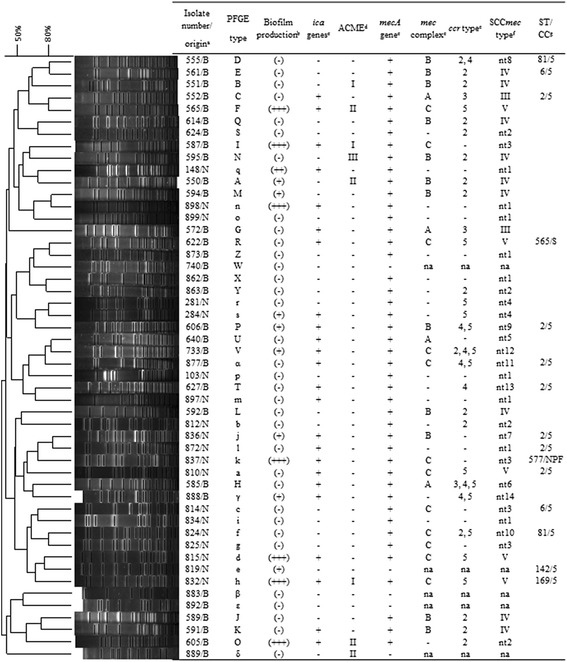
Dendrogram of the pulsed-field gel electrophoresis (PFGE) profiles of *Sma*I-digested genomic DNA of 50 genetically unrelated *Staphylococcus epidermidis* isolates and associated characteristics. Similarities percentage is identified on a dendrogram derived from the unweighted pair group method using arithmetic averages and based on Dice coefficients. ^a^ B: Bloodstream infection isolates, N: Nasal isolates; ^b^ (+++): strong; (++): moderate; (+): weak: (**−**): non-biofilm producer; ^c^ +: presence; −: absence; ^d^ I: *arcA*+/*opp3AB*+, II: *arcA*+/*opp3AB*-, III: *arcA*-/*opp3AB*+; −: negative; ^e^ −: not-detectable; na: not-applicable (methicillin-sensitive isolate); ^f^ na: not-applicable (methicillin-sensitive isolate); nt: non-typeable; ^g^ ST: sequence type; CC: clonal complex; S: singleton; NPF: none predicted founder

One hundred twenty-six *S. epidermidis* isolates from 126 neonates admitted in NICUs of four hospitals at Rio de Janeiro, Brazil, between May 2007 and March 2012 and belonging to the laboratory collection were characterized. Among them, 54 were recovered from blood cultures and related to bloodstream infections (BSIs) following the Center of Disease Control (CDC) criteria (2008). The other 72 isolates were obtained from nasal swabs by the infection control commission professionals. One isolate per patient was included in this study. After identification of all isolates as *S. epidermidis* by the simplified phenotypic [22] and PCR methods [23], they were characterized by pulsed field gel electrophoresis (PFGE) to exclude the clonality of isolates. Bacterial DNA was extracted and digested with the *SmaI* enzyme [24] and the restriction fragments were separated using a BioRad CHEF DR III apparatus, the PFGE profiles obtained were analyzed with Bio-Numerics software. Similarity percentage was identified on a dendrogram derived from the unweighted pair group method using arithmetic averages and based on Dice coefficients. Isolates showing a similarity coefficient < 80% or differences of five or more bands were considered genetically unrelated. For the 126 *S. epidermidis* isolates evaluated 50 different PFGE genetic backgrounds were identified, corresponding to 31 BSI isolates and 19 isolates from nares (Fig. 1). One representative isolate of each PFGE genotype was randomly selected for this study.

### *mecA* gene detection and SCC*mec* typing

Bacterial DNA was extracted as previously described by Pitcher [25] and then detection of the *mecA* gene and SCC*mec* typing were performed according with Del Vecchio et al. and Kondo et al., respectively [26, 27]. The latter method consists of two multiplex PCR to detect the *ccr* complex (encoding for recombinases) and the *mec* complex (encoding for beta-lactam resistance). The combination of the types of *ccr* and *mec* class allowed the identification of the type of SCC*mec* (I to IX), and verify non-typeable isolates. The following *S. aureus* reference strains and clinical isolates were used as positive controls for SCC*mec* typing: EMRSA-3/Cordobés (SCC*mec* I) [28]**,** Mu50 (SCC*mec* II) [29], HU25 (SCC*mec* III) [24], 527a (SCC*mec* IV) [30] and 557a (SCC*mec* V) [31].

### Phenotypic detection of biofilm formation

Biofilm formation was determined according to Iorio et al. [18]. The *S. epidermidis* strains ATCC 35984 (formerly RP62A) and ATCC 12228 were used as positive and negative controls, respectively. All isolates were classified into the following categories: strong, moderate, weak and non-biofilm producer.

### PCR assays for biofilm associated genes and ACME elements

The detection of the virulence genes *aae*, *atlE*, *aap*, *bhp*, *embp*, *fbe*, *gehD*, *sdrF, sesI*, the *icaADB* operon and ACME allotypes was performed by PCR. ACME allotypes were classified as: ACME-I contains both the *arc* and the *opp-3* gene clusters; ACME-II contains *arc* but not *opp-3*; and ACME-III contains *opp-3* without *arc* [20]. Bacterial DNA was extracted as previously described [25]. The primers and PCR conditions are summarized in the Table 1. *Staphylococcus* spp. reference strains used as positive controls were *S. epidermidis* ATCC 35984 (*aae*, *aap*, *bhp*, *embP*, *gehD*, *icaABD* and *sesI* genes), *S. epidermidis* ATCC 12228 (*sdrF* gene), *S. epidermidis* ATCC 14490 (*atlE* and *fbe* genes) and a clinical isolate of *S. aureus* number 526a/USA300 (*arcA* and *opp3AB* genes) [30].

**Table 1:** Genes, oligonucleotide primers and PCR conditions used in this study

Protein or genetic element	Gene	Primers 5’ → 3’^a^	Amplicon size (bp^b^)	PCR conditions	References
Aae	*aae*	F: GAGGAGGATTTTAAAGTGC R: AACATGACCATAGTAACC	858	94 °C, 3 min; 40 cycles of: 94 °C, 90s; 55 °C, 1 min; 72 °C, 90s; final extension 72 °C, 5 min.	[8]
Aap	*aap*	F: ATACAACTGGTGCAGATGGTTG R: GTAGCCGTCCAAGTTTTACCAG	400	94 °C, 3 min; 30 cycles of: 94 °C, 1 min; 50 °C, 1 min; 72 °C, 1 min; final extension 72 °C, 5 min.	[52]
AtlE	*atlE*	F: CAACTGCTCAACCGAGAACA R: TTTGTAGATGTTGTGCCCCA	682	94 °C, 3 min; 30 cycles of: 94 °C, 1 min; 62 °C, 1 min; 72 °C, 1 min; final extension 72 °C, 5 min.	[33]
Bhp	*bhp*	F: ACGGACAATATCGTCTCTCAA R:: AACTTCAGCCGTTCCCTT	1917	94 °C, 2 min; 40 cycles of: 94 °C, 30s; 55 °C, 30s; 72 °C, 75 s; final extension 72 °C, 5 min.	[10]
Embp	*embp*	F: AGCGGTACAAATGTCAATATC R: AGAAGTGCTCTAGCATCATCC	455	94 °C, 3 min; 30 cycles of: 94 °C, 1 min; 62 °C, 1 min; 72 °C, 1 min; final extension 72 °C, 5 min.	[6]
GehD	*gehD*	F: TTTGAATTCTGCGCAAGCTCAATATAA R: TTTGCGGCCGCTATCGCTACTTACGTGTAA	1179	94 °C, 2 min; 30 cycles of: 94 °C, 30s; 55 °C, 30s; 72 °C, 75 s; final extension 72 °C, 5 min.	[9]
SdrF	*sdrF*	F: GCTGAAGACAATCAATTAG R: TTAATATCCCCCTGTGCTG	1875	94 °C, 4 min; 30 cycles of: 94 °C, 2 min; 60 °C, 1 min; 72 °C, 2 min; final extension 72 °C, 5 min.	[10]
SdrG	*fbe*	F: TAAACACCGACGATAATAACCAAA R: GGTCTAGCCTTATTTTCATATTCA	496	94 °C, 3 min; 30 cycles of: 94 °C, 1 min; 62 °C, 1 min; 72 °C, 1 min; final extension 72 °C, 5 min.	[5]
SesI	*sesI*	F: GCTGATTATGTAAATGACTCAAAT R: AGCTTTTGTTGTTTGAGCTTC	408	94 °C, 3 min; 30 cycles of: 94 °C, 1 min; 50 °C, 1 min; 72 °C, 1 min; final extension 72 °C, 5 min.	[11]
IcaADB	*icaADB*	F: TTATCAATG CCGCAGTTGTC R: GTTTAACGCGAGTGCGCTAT	546	94 °C, 3 min; 30 cycles of: 94 °C, 1 min; 58 °C, 1 min; 72 °C, 1 min; final extension 72 °C, 5 min.	[33]
ACME	*arcA*	F: CTAACACTGAACCCCAATG R: GAGCCAGAAGTACGCGAG	1946	95 °C, 10 min; 30 cycles of: 94 °C, 1 min; 52 °C, 1 min; 72 °C, 2 min; final extension 72 °C, 5 min.	[43]
	*opp3AB*	F: GCAAATCTGTAAATGGTCTGTTC R: GAAGATTGGCAGCACAAAGTG	1183		

^a^ F: Forward; R: Reverse

^b^ bp: base pairs

### Multilocus sequence typing

Among the 126 *S. epidermidis* isolates previously analyzed by PFGE, 15 isolates (seven BSI and eight nasal) that represented genotypes clustering five or more isolates were selected for characterization by MLST in the present study [32]. The PCR products were purified using the commercial system “GTX PCR and band purification” (GE 50 Healthcare, Buckinghamshire, England) according to the manufacturer’s specifications. The purified products were sequenced using the automated DNA sequencer ABI3100 (Applied Biosystems, Foster, CA, USA). Sequence types (ST) were determinate using the MLST database (http://www.mlst.net/) and characterized as singletons or members of a clonal complex (CC) by the eBURST algorithm (accessible at http://eburst.mlst.net/). Numbers for new ST reported here were assigned by the *S. epidermidis* MLST database curator.

### Statistical methods

All comparisons were performed using the *χ* ^2^ test or the Fischer’s exact test. Differences were considered statistically significant when values of *p* < 0.05 were obtained.

## Results

### Biofilm production and genes associated

The ability to produce biofilm was analyzed for 50 *S. epidermidis* neonatal isolates from BSI (31 isolates) and nasal colonization (19 isolates). Biofilm formation was positive for 16 isolates, 8 (25.8%) from BSI and 8 (42.1%) from nares (Table 2); no significant difference between the groups was observed. Among the BSI isolates, three were classified as strong and five as weak biofilm producers. Among nasal isolates, four were strong, one moderate and three were weak biofilm producers. All the *S. epidermidis* isolates, irrespective to the biofilm production, carried at least three of the ten biofilm-associated genes investigated in this study, and 86% of them harbored five or more of these genes. The *icaADB* genes were present in 81.2% of the biofilm producer isolates and were detected in all isolates classified as moderate and strong producers and in the majority (5/8; 62.5%) of the weak biofilm producers. These genes were detected in 14 (45.2%) of the BSI and 10 (52.6%) of the nasal isolates. The *aae*, *atlE*, *embp* and *fbe* genes were frequently found in both groups of isolates, ranging from 74 to 100%. However, the *sdrF* and *sesI* genes were more commonly found among BSI isolates (*p* = 0.001 and *p* = 0.02, respectively). The *sdrF* gene was also significantly more associated with the biofilm producer isolates from BSI (*p* = 0.007). Furthermore, the presence of the *aap* gene was more frequent among biofilm producer isolates (15/16; 93.7%) than among non-biofilm producers (19/34; 55.9%) (*p* = 0.009).

**Table 2:** Virulence genes and biofilm production in 50 *Staphylococcus epidermidis* isolates from bloodstream infection and nasal colonization

Biofilm-associated virulence genes	Number (%) of isolates			Number (%) of biofilm producer isolates		
	BSI^a^ (*n* = 31)	Nasal^b^ (*n* = 19)	*p* value	BSI^a^ (*n* = 8)	Nasal^b^ (*n* = 8)	*p* value
*aae*	31 (100)	19 (100)	1	8 (100)	8 (100)	1
*aap*	18 (58)	16 (84)	0.068	7 (88)	8 (100)	1
*atlE*	30 (97)	19 (100)	1	8 (100)	8 (100)	1
*bhp*	1 (3)	1 (5)	1	0 (0)	0 (0)	1
*embp*	28 (90)	19 (100)	0.279	8 (100)	8 (100)	1
*fbe*	23 (74)	16 (84)	0.498	7 (88)	6 (75)	1
*gehD*	23 (74)	11 (58)	0.349	6 (75)	6 (75)	1
*sdrF*	20 (65)	2 (11)	0.001^c^	6 (75)	0 (0)	0.007^c^
*sesI*	12 (39)	0 (0)	0.02^c^	3 (38)	0 (0)	0.2
*icaADB*	14 (45)	10 (53)	0.772	6 (75)	7 (88)	0.6
*arcA*	6 (19)	1 (5)	0.229	4 (50)	1(13)	0.282
*opp3AB*	3 (10)	1 (5)	1	1 (13)	1 (13)	1

^a^ BSI: Bloodstream infection isolates

^b^ Nasal: Nasal isolates

^c^: results with statistical significance

Taken together, the detection of all biofilm associated genes showed 28 different genetic profiles (Table 3). Seven of them are shared by isolates of both groups and included 22 (44%) isolates. Seventeen profiles were exclusive for the BSI isolates and four for the nasal colonizers. The majority of the profiles (78.6%) included only 1 or 2 *S. epidermidis* isolates, demonstrating a wide diversity of virulence genes profiles in this staphylococci species.

**Table 3:** Virulence genes profiles and SCC*mec* types identified among 50 *Staphylococcus epidermidis* isolates from bloodstream infection and nasal colonization

*S. epidermidis* isolates source (*n*)^a^	Isolates	Biofilm-associated virulence genes ^c^										SCC*mec* types (*n*) ^b, d^
	(*n*)^b^	*icaADB*	*aae*	*aap*	*atlE*	*bhp*	*embp*	*fbe*	*gehD*	*sdrF*	*sesI*	
BSI and Nasal (22)	6 (1B + 5 N)	+	+	+	+	-	+	+	+	-	-	V (1B + 2 N), nt1, nt3, nt7
	4 (1B + 3 N)	-	+	+	+	-	+	+	+	-	-	IV (1B), nt1, nt10, na
	3 (2B + 1 N)	-	+	+	+	-	+	+	-	-	-	IV (1B), nt3, na
	3 (1B + 2 N)	+	+	+	+	-	+	+	-	-	-	nt3 (1B), nt1 (2 N)
	2 (1B + 1 N)	-	+	-	+	-	+	+	+	-	-	IV (1B), nt3
	2 (1B + 1 N)	+	+	+	+	-	+	+	+	+	-	nt2 (1B), V
	2 (1B + 1 N)	-	+	+	+	-	+	-	-	-	-	nt2 (1B), nt4
BSI (23)	4	+	+	+	+	-	+	+	+	+	+	III, nt5, nt9, nt12
	3	-	+	+	+	-	+	+	+	+	+	IV (2), nt8
	2	-	+	+	+	-	+	+	+	+	-	IV, na
	1	+	+	-	+	-	+	+	+	+	+	III
	1	-	+	+	+	-	+	+	+	-	+	IV
	1	+	+	-	+	-	+	+	+	+	-	IV
	1	-	+	-	+	-	+	+	+	+	+	IV
	1	+	+	-	+	-	+	-	+	-	-	V
	1	-	+	-	+	-	+	-	-	+	+	nt2
	1	+	+	-	+	-	-	+	+	+	+	nt6
	1	+	+	-	+	-	-	+	-	-	-	nt13
	1	-	+	-	+	+	+	-	+	+	-	na
	1	-	+	-	+	-	+	-	+	-	-	nt1
	1	-	+	-	+	-	+	-	+	+	-	nt1
	1	+	+	+	+	-	+	+	-	+	-	nt11
	1	-	+	-	-	-	-	-	+	+	-	na
	1	+	+	-	+	-	+	-	-	+	-	nt14
Nasal (5)	2	-	+	-	+	-	+	+	-	-	-	nt1
	1	+	+	+	+	-	+	-	-	-	-	nt4
	1	+	+	+	+	-	+	-	+	-	-	nt1
	1	-	+	+	+	+	+	+	-	+	-	nt2

^a^ BSI: Bloodstream infection isolates

^b^ B: Bloodstream infection isolates; N: Nasal isolates

^c^ +: presence; -: absence

^d^ III: *mec* complex A/*ccr* 3; IV: *mec* complex B/*ccr* 2; V: *mec* complex C/*ccr* 5; nt: non-typeable; na: not-applicable (methicillin-sensitive isolate); nt1: *mec* complex -/*ccr* -; nt2: *mec* complex -/*ccr* 2; nt3: *mec* complex C/*ccr* -; nt4: *mec* complex -/*ccr* 5; nt5: *mec* complex A/*ccr* -; nt6: *mec* complex A/*ccr* 3,4 and 5; nt7: *mec* complex B/*ccr* -; nt8: *mec* complex B/*ccr* 2 and 4; nt9: *mec* complex B/*ccr* 4 and 5; nt10: *mec* complex C/*ccr* 2 and 5; nt11: *mec* complex C/*ccr* 4 and 5; nt12: *mec* complex C/*ccr* 2,4 and 5; nt13: *mec* complex -/*ccr* 4; nt14: *mec* complex -/*ccr* 4 and 5

### ACME detection

Among the BSI isolates two of them harbored the *arcA* and *opp3AB* genes (ACME I), four had only the *arcA* gene (ACME II) and one had only the *opp3AB* (ACME III) (Table 2). ACME elements were detected only in one nasal isolate, corresponding to ACME I. Despite the frequent presence of the ACME elements among the BSI isolates (seven isolates), no statistical significance was verified. Four of seven strong biofilm producer isolates harbored the ACME, however no association between the presence of this genetic island and biofilm production (*p* = 0.092) or the strong biofilm production (*p* = 0.106) was detected. Of eight ACME positive isolates, five were included in SCC*mec* types IV (three isolates) or V (2).

### Detection of the *mecA* gene and SCC*mec* typing

The *mecA* gene was detected in 27/31 (87.1%) and 18/19 (94.7%) of the BSI and nasal isolates, respectively. For the 27 methicillin-resistant *S. epidermidis* (MRSE) isolates from BSI that were analyzed for composition of SCC*mec* elements, 13 (48.1%) were typeable: 9 (69.2%) harbored the SCC*mec* type IV, 2 (15.4%) the type III and 2 (15.4%) the type V (Fig. 1, Table 3). Many of the isolates from BSI (14/51.9%) were classified as non-typeable (nt), which possessed more than one *ccr* allotype; no *ccr* allotype or *mec* complex detectable; or no *ccr* allotype and *mec* complex detectable. Among the 18 MRSE nasal isolates only 3 (16.7%) were classified into a SCC*mec* type and harbored the type V, whereas the other 15 (83.3%) isolates were nt.

While the BSI isolates harbored more frequently the SCC*mec* type IV (33.3%) (*p* = 0.007) or the *ccr* complex 2 (51.9%) (*p* = 0.013), the nasal isolates showed an undetectable *ccr* complex (11 isolates; 61.1%) (*p* = 0.003) that included seven isolates of the prevalent nt1 profile (no *mec* complex and no *ccr* detectable) (*p* = 0.019).

### Diversity and MLST characterization

The dendrogram obtained for the 50 *S. epidermidis* evaluated showed a distribution of isolates in seven larger clusters and three of them were composed exclusively by BSI or nasal isolates (Fig. 1). It was also possible identify eight pair of isolates that group together with about 80% of similarity. Among them the isolates of each of the pairs 555-561 and 552-565 belonged to BSI isolates from the same hospital and were recovered with about four months of difference. Although of this fact the isolates presented different genetic and phenotypic characteristics, including composition of the SCC*mec* elements, ST and virulence genes. The isolates of each of the pairs 836-872, 281-284 and 824-825 belonged to the same clinical origin (nasal) and NICU and had similar characteristics between each of them, but they showed differences in the composition of their SCC*mec* types or virulence. The remaining three pairs of isolates (622-873, 640-733 and 594-898) were from different NICUs and periods of isolation, and presented remarkable differences between the isolates within the pairs.

For seven BSI isolates evaluated by MLST, 4 STs were identified: ST2 (four isolates), ST6, ST81 and ST565 (Fig. 1). Among the eight nasal isolates evaluated, ST2 was also the most frequent (three isolates), whereas the STs 6, 81, 142, 169 and a new ST577 were also identified. The majority (13/15) of the isolates analyzed were included into CC5, the major CC of *S. epidermidis*. Two *S. epidermidis* isolates were not classified into CC5, the ST577 with no predicted founder and a singleton ST565.

## Discussion

Several extrinsic factors associated with the nosocomial environment may disturb the delicate host-microbe balance of the neonates, resulting in a lifestyle conversion of *S. epidermidis* from mutualism to pathogenicity [2]. This species has become the focus of studies that attempt to understand which bacterial features can help the establishment of such infections. In this study, we evaluated different molecular characteristics associated with virulence and resistance in 50 genetically unrelated *S. epidermidis* isolates from neonates, 31 from BSIs and 19 from nasal colonization. We found some characteristics that differentiate isolates of these two groups, showing that some aspects could provide advantages to the pathogen to increase its colonization and pathogenicity.

Some authors have proposed that the ability to produce biofilm in combination with the presence of the *ica* operon could be used as pathogenesis markers to distinguish invasive from commensal isolates [33, 34]. Our results and other studies [35, 36] demonstrated no significant differences on biofilm production or presence of the *ica* genes between isolates of both groups. On the other hand, it should be noted that only 16 isolates of this study showed phenotypically biofilm production, while 45% of the BSI and 53% of the nasal isolates harbored the *ica* genes. It is possible that this fact was changed by addition of supplementary factors to the culture medium, such as glucose or NaCl, which could lead to or increase biofilm production [37, 38].

This is the first report to show a differentiated distribution of the biofilm-associated *sdrF* gene, found almost exclusively in BSI isolates. In order to confirm this result other 40 nasal isolates were also tested and only 25% were positive for the gene, maintaining a significant difference in relation to invasive isolates. Some studies have demonstrated that the SdrF protein is able to mediate alone the adherence of *S. epidermidis* to a wide variety of plastic materials, such as catheters and other prosthetic devices, participating in the initial adhesion through ionic interactions and then to the collagen, through specific receptor-ligand interactions [4, 39]. Taking this into consideration, *S. epidermidis* isolates carrying the *sdrF* gene would have a greater potential to attach to medical devices, aspect considerably advantageous for the establishment of invasive infections, including BSI.

Similarly to our results, Söderquist and coworkers [11] found a significant association between the presence of the *sesI* gene in invasive isolates and its absence among commensal ones, suggesting that this gene may be a possible virulence marker. To confirm these results, we tested other 40 isolates from neonate nares and found only two *S. epidermidis* isolates carrying *sesI* gene among them (data not shown), reinforcing the near absence of this gene among commensal isolates. However, Bowden and coworkers [10] already had detected the *sesI* gene in 45% of the *S. epidermidis* isolates from neonates BSI and in 29% of the contaminants isolates, as well as in 34% of the skin isolates from these patients. According to Söderquist et al. [11] since healthy people did not regularly carry *sesI* positive isolates the patients could acquire these isolates from the hospital environment after admission and become colonized. In addition, invasive *S. epidermidis* isolates can be selected during hospitalization, and this period responsible for changing a commensal bacterial population by isolates with greater virulence. However, the correlation of this gene with pathogenicity is not clear yet and further studies are necessary to understand the contribution of the SesI protein to the pathogenic potential of *S. epidermidis*.

In this study the presence of the *aap* gene was associated with biofilm producer isolates, and this association may be explained considering the important functions of the Aap protein in adhesion and intercellular aggregation, allowing the biofilm formation. In this line, two studies involving *S. epidermidis ica* operon deficient isolates identified the Aap protein as the major adhesin responsible for biofilm composed mainly of proteinaceous factors [13, 40]. This fact could explain how three *ica* operon-deficient isolates but *aap* gene-positive from the present study were able to produce biofilm, supporting the important role of this gene on *ica*-independent biofilm formation. Concerning the presence almost ubiquitous of the *atlE*, *aae*, *fbe* and *embp* genes, as well as the low frequency of the *bhp* gene observed in this study, other authors had also found similar results in *S. epidermidis* isolates [10, 33–35, 41].

The acquisition of the genetic island ACME by the staphylococci species seems to provide advantages in terms of host colonization, rather than an enhanced pathogenicity [20]. In the present study, only 8 (16%) of the 50 isolates harbored this element, which do not support other studies that found a high prevalence of ACME elements among *S. epidermidis* isolates [20, 42]. Svensson and coworkers [43] evaluated *S. epidermidis* isolates from neonates and detected this genetic element in 43% of blood isolates, while Granslo et al. [44] detected it in 23% of blood isolates, similarly to our results in relation to the BSI isolates (7/31; 22.6%). However, these authors also detected ACME in many isolates considered as contaminants and they concluded that this genetic element do not seem to be associated with increased pathogenicity of *S. epidermidis*. Geographic location could interfere in its occurrence, but additional studies are needed to clarify these findings. It has also been proposed that the *ccrAB* complex of the SCC*mec* element could be implicated in ACME mobilization [20]. However, in our study no association was found between any SCC*mec* type or *ccr* complexes and the ACME elements among the *S. epidermidis* isolates analyzed.

The high proportion of BSI *S. epidermidis* isolates harboring the *mecA* gene has become an expected fact. In our study, this gene was detected in 94.7% of the nasal isolates, which contrasts with the low frequencies described by Cherifi and coworkers [45] for their set of commensal isolates. However, it is important recognize the difference between commensal isolates from healthy individuals, without contact with a hospital and those obtained from hospitalized patients, as described by Rohde and coworkers [35]. These authors found great similarities in relation to the detection of the *mecA* gene and virulence factors between invasive and commensal isolates from patients with bone marrow transplantation, but with a striking difference from those obtained from healthy volunteers. This can be explained by the selective pressure that the hospital environment exerts, thus could led to the acquisition of genetic elements, including resistance and/or virulence genes, which can ensure the survival of the microorganism.

Concerning the SCC*mec* types, it was observed that the type IV was prevalent in BSI isolates (*p* = 0.007), while the type III was detected only in two of them, in contrast with the study of Pinheiro and coworkers [46] that detected 53.2% of their MRSE isolates from blood cultures carrying the SCC*mec* type III. However, the high frequency of the SCC*mec* IV in clinical isolates of *S. epidermidis* was already observed previously [47–49], demonstrating that this SCC*mec* type has become a common occurrence among hospital isolates. In addition, it was observed in our study that the *ccr2* complex that comprises the SCC*mec* type IV was also associated with BSI isolates. Similarly, Svensson and coworkers [43] also reported a major frequency of *ccr2* complex in their set of *S. epidermidis* isolates from blood cultures of neonates, while, Barbier et al. [42] found this association type with isolates recovered from nares. These data suggest that *S. epidermidis* appear to be efficient to acquire the *ccr2* complex and could be the major reservoir for this type of genetic element, regardless of the origin of isolates.

A high proportion of isolates was SCC*mec* non-typeable (64.4% in this study) and this finding was not surprising, since this fact has been frequently reported among CNS isolates, independent of the clinical origin [36, 43, 48, 50]. However, in our study non-typeable profiles were more frequently associated with nasal isolates (83.3%), including the nt1 prevalent profile (*ccr*-*mec* complex undetectable). These data show that *S. epidermidis* isolates from the nasal origin can present a high genetic diversity of the SCC*mec* elements. This diversity was shown by Conlan and coworkers [51] who observed that *S. epidermidis* commensal isolates have an open pan-genome with considerable diversity between isolates, even when derived from a single individual or body site.

In this study, the MLST results for some selected isolates showed that the ST2 was the most frequent lineage, independent of the clinical origin. Moreover, this ST is included into the CC5 that was designated for 13 of 15 *S. epidermidis* isolates analyzed. ST2 has been reported to be the most widely disseminated hospital-associated ST type among *S. epidermidis* isolates [17, 18, 47, 51]. According to Li et al. [17], the successful spread of this lineage may be associated with the fact that, by recombination, ST2 generates novel phenotypic and genotypic variants, such as *ica* genes-positive isolates, which makes ST2 isolates easily able to spread in the hospital environment.

The greatest limitation of this study is the small number of isolates tested. In order to have as much diversity as possible and the results obtained be not biased since some isolates belonged to the same clonal group, the final selection based on the results of PFGE greatly reduced this number. However, for the virulence genes that had demonstrated significant differences, an extra number of isolates was tested and the results found were confirmed. On the other hand, the inclusion of nasal *S. epidermidis* isolates from healthy volunteers without any relation to the hospital environment could give us an overview of the presence of the virulence genes, such as *sdrF*, to confirm the importance of this gene as a significant marker.

## Conclusions

This study showed that despite the great clonal diversity displayed by the *S. epidermidis* isolates from neonates, those from BSI harbored more frequently the *sdrF* and *sesI* biofilm-associated genes. Moreover, mostly of BSI isolates carried the SCC*mec* type IV or the *ccr*2 complex, while 83.3% of nasal isolates were non-typeable, showing more diversity for the SCC*mec* elements composition. It is interesting to note that even being our set of *S. epidermidis* isolates from patients in contact with the hospital environment we found significant differences in two genes that eventually could be used as markers of invasiveness. However, *S. epidermidis* possesses a great genetic plasticity that allow acquire, lose or regulate genetic elements that provide advantages to improve its colonization in the host increasing its pathogenicity.

## Abbreviations

Aae: Autolysin/adhesin of *S. epidermidis*
Aap: Accumulation-associated protein
ACME: Arginine catabolic mobile element
ATCC: American Type Culture Collection
AtlE: Autolysin of *S. epidermidis*
B or BSI: Bloodstream infection
Bhp: Bap homologue protein
bp: Base pairs
CC: Clonal complex
CDC: Centers for Disease Control and Prevention
CNS: Coagulase negative staphylococci
Embp: Extracellular matrix-binding protein
F: Forward
GehD: Extracellular lipase of *S. epidermidis*
MLST: Multilocus sequence typing
MRSE: Methicillin-resistant *Staphylococcus epidermidis*
MSCRAMMS: Microbial surface components recognizing adhesive matrix molecules
N: Nasal
na: Not-applicable
NICUs: Neonatal intensive care units
NPF: None predicted founder
nt: Non-typeable
PBP: Penicillin-binding protein
PCR: Polymerase chain reaction
PFGE: Pulsed field gel electrophoresis
PIA: Polysaccharide intercellular adhesin
R: Reverse
SCC*mec*: Staphylococcal chromosomal cassette *mec*
SdrF: Serine/aspartate repeat protein F
SdrG/Fbe: Serine/aspartate repeat protein G/fibrinogen-binding protein
SesI: *S. epidermidis* surface protein I
ST: Sequence type
